# Edible Insects an Alternative Nutritional Source of Bioactive Compounds: A Review

**DOI:** 10.3390/molecules28020699

**Published:** 2023-01-10

**Authors:** Donatella Aiello, Marcella Barbera, David Bongiorno, Matteo Cammarata, Valentina Censi, Serena Indelicato, Fabio Mazzotti, Anna Napoli, Daniela Piazzese, Filippo Saiano

**Affiliations:** 1 Department of Chemistry and Chemical Technologies, University of Calabria, 87036 Arcavacata di Rende, Italy; 2 Department of Earth and Marine Sciences, University of Palermo, 90123 Palermo, Italy; 3 Department of Biological, Chemical and Pharmaceutical Science and Technology (STEBICEF), University of Palermo, 90123 Palermo, Italy; 4 Department Agricultural Food and Forestry Sciences, University of Palermo, 90128 Palermo, Italy

**Keywords:** insect-based foods, polyphenols, Folin–Ciocalteu method, phenols and flavonoids, polyphenols bioactivity

## Abstract

Edible insects have the potential to become one of the major future foods. In fact, they can be considered cheap, highly nutritious, and healthy food sources. International agencies, such as the Food and Agriculture Organization (FAO), have focused their attention on the consumption of edible insects, in particular, regarding their nutritional value and possible biological, toxicological, and allergenic risks, wishing the development of analytical methods to verify the authenticity, quality, and safety of insect-based products. Edible insects are rich in proteins, fats, fiber, vitamins, and minerals but also seem to contain large amounts of polyphenols able to have a key role in specific bioactivities. Therefore, this review is an overview of the potential of edible insects as a source of bioactive compounds, such as polyphenols, that can be a function of diet but also related to insect chemical defense. Currently, insect phenolic compounds have mostly been assayed for their antioxidant bioactivity; however, they also exert other activities, such as anti-inflammatory and anticancer activity, antityrosinase, antigenotoxic, and pancreatic lipase inhibitory activities.

## 1. Introduction

**Figure 1:**
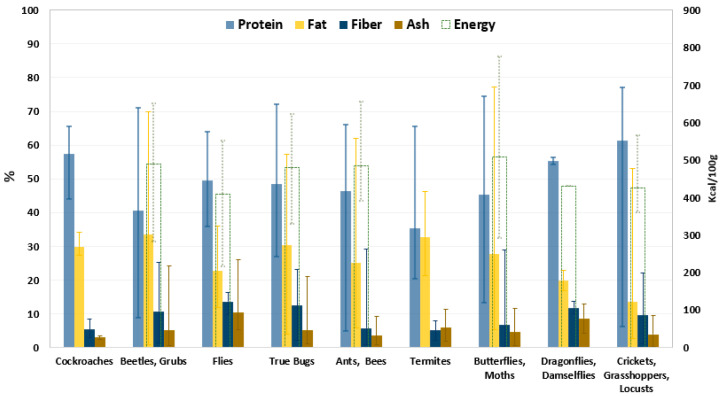
Nutritional composition [%] and energy content [kcal/100 g] of edible insects. Data are reported as average value of dry matter and the error bars indicate the maximum and minimum value determined [6].

The consumption of edible insects has been a food habit for thousands of years and is common in 120 countries around the world [1,2]. Around 2000 edible insect species raw or processed are consumed across Asia, Australia, Africa, and Central and South America, while insect consumption is uncommon in Western societies [3,4]. The most widespread insect species in the world are beetles followed in descending order by caterpillars; ants, bees, and wasps; grasshoppers and locusts; true bugs; dragonflies; termites; flies; and cockroaches [3,5]. Due to the wide range of edible insect species, their nutritional value is highly variable: A summary of the nutrient composition of more than 200 edible insects (based on dry matter) is shown in Figure 1 [6]. It is possible to observe that the composition of edible insects is generally subject to great variation even within the same species. For example, the species of the order Orthoptera, including crickets, grasshoppers, and locusts, have an average protein content of 61% with variations ranging from 6 to 77%. This suggests that this variation not only result from differences between species and developmental stages but also from different feed and geographical origins, as well as differences in measuring methods. However, globally, the most common edible insects are rich in protein, mono- and polyunsaturated fats, and fiber [6,7,8].

This aspect leads several international agencies, such as the Food and Agriculture Organization (FAO), to consider insects as cheap, highly nutritious, and healthy food sources [9,10]. In addition, insects have become important in traditional Oriental medicine as a regular treatment for gastritis, fever, cough, asthma, arthritis, rheumatism, and diabetes [6,11,12,13]. Due to their recognized pharmacological properties, scientific research has focused on the beneficial properties of insects for human health. Recent studies report that edible insects can provide bioactive compounds, such as phenolic compounds and flavonoids [7,12,14,15,16,17], acting as antioxidant, anti-inflammatory, anticancer, antimicrobial, and antibacterial inhibitors of the pancreatic lipase enzyme, insulin regulators, and glycaemic inhibitors [13,18,19,20,21,22,23]. Although beneficial effects are often due to the synergy of different components, several studies report the key role of polyphenolic content concerning specific bioactive activities. For instance, experimental in vitro studies demonstrated the antioxidant effect of polyphenolic compounds derived from the extracts of house crickets (*Acheta domesticus*), mealworms (*Tenebrio molitor*) [24], and dark black chafer beetles (*Holotrichia parallela*) [14]. An in vivo study performed in mice [25] proved the antioxidant effects of phenolic compounds in a vegetal tea and an insect tea (*Hydrillodes repugnalis*). They found that mice treated with insect tea showed higher superoxide dismutase, glutathione peroxidase, glutathione activities, and lower nitric oxide and malonaldehyde activities than control group mice. In addition to antioxidant properties, it has been reported that polyphenols from the hydroethanolic extracts of the edible insect *Polyrhachis vicina* may act as pancreatic lipase inhibitors [22]. Moreover, other authors [26] reported that hydroxytyrosol dimers isolated from *Brynchoptera rynchopetera* exhibited selective cytotoxicity and good inhibitory activity on mouse melanoma proliferation. In addition to the nutritional benefits, a new global interest in edible insects and invertebrates has recently emerged as the imbalance between the production and consumption of animal-origin food is increasing. This is creating various socioeconomic concerns, exacerbated by climate change [10,27]. From an environmental and ecological point of view, insects represent a highly sustainable replacement for meat and animal products. Their farming results in lower greenhouse gas emissions, requires less water and land, represents a much lower economic investment compared to livestock, and also can limit deforestation for pasture use (Table 1) [28].

From a social and economic perspective, the insect market offers livelihood and entrepreneurial development opportunities for farmers in rural communities, thus improving the quality of life in poor and developing countries [9,34,35,36,37]. Furthermore, primary production and processing of edible insects are expected to increase in Western countries, resulting in new commercial opportunities and new sources of income [6,10,38]. Despite all these favorable aspects, the consumption of edible insects has been associated with several risk factors, including biological, toxicological, and allergenic risks [8,10,39,40,41,42]. Molds and yeasts with mycotoxigenic potential can affect edible insects causing adverse reactions in humans [8,43,44]. The presence of tropomyosin in insects, a fibrous protein also found in crustaceans and arthropods, can cause allergic reactions in consumers [45,46]. Microbial contamination can also affect insects, but with a higher risk for wild ones [47]. Further, standards or criteria that determine guidelines for management and hygiene are still fragmented, increasing consumer mistrust, especially in Western countries where entomophagy is not fully accepted [10,42,48]. Globally, rules and regulations, both at the national and international level, on the production, storage, and consumption of insects as food/feed ingredients are often absent or, at best, nonexhaustive [4,28,42,49]. Although cultural barriers and legislative deficits related to entomophagy still exist, the human consumption of insects is becoming strategic due to their health properties, high nutritional values, and environmental sustainability [10,38]. Currently, research trends and food innovations focus on food fortification with alternative, sustainable, and functional food to improve the nutritional value of food by correcting nutrient or minerals deficiencies or enhancing health-promoting properties [50,51,52,53,54,55,56,57,58]. In this contest, insects represent one of the most valuable functional ingredients and interesting solutions for the food industry [13,59,60,61,62,63]. Insect powder can be a valuable ingredient to supplement deficiencies in food, such as in gluten-free products. For example, the enrichment of gluten-free bread with 10% of cricket powder significantly increased the nutritional value, with protein content exceeding seven-fold the reference bread [64]. Moreover, cricket powder addition resulted in an increase of the total polyphenolic compound content from 1.9 mg/g in control bread to 6.2 mg/g in insect-enriched bread; likewise, the total antioxidant capacity before digestion increased about fourfold and after digestion about sixfold without affecting either beneficial or pathogenic microflora. Zielinska et al. [65] tested the effect of adding cricket (*Gryllodes sigillatus*) and mealworm (*T. molitor*) flours to muffins. In muffins enriched with insect flour, they found an increase in protein content, a decrease in carbohydrate content, and a reduction in the glycemic index. In addition, the antioxidant capacity, as well as total phenolic content, increased correspondingly as the percentage of insect flour in the muffins increased. Additionally, enriched muffins were accepted by consumers. The researcher also reported that the use of appropriate processes in food technology can ensure high retention of nutrients and bioactive compounds in food, enhancing its health properties [66]. For instance, Gaglio et al. [67] investigated in vitro the antioxidant potential of bread added with mealworm and buffalo worm before and after gastrointestinal digestion in comparison with insect-free bread. The authors found that replacing semolina with buffalo and mealworm powder increased the antioxidant capacity of the bread before digestion by three- and four-fold, respectively. After gastrointestinal digestion, all samples (bread with insects and control bread) showed a higher antioxidant capacity value than that measured before digestion. This may be explained because phenolic antioxidants can be released from the wheat during digestion, increasing the antioxidant potential of the digested bread [67]. Interesting to note is that, in other studies, fortification of bread with plant-based additives, such as Saskatoon berry powder or grape pomace, resulted in a similar increase in the antioxidant potential of the product [68,69,70]). The increased polyphenol content in fermented samples could be explained because, during the fermentation process, bacteria may remove sugar moieties, hydrolyze galloyl moieties, and release free phenolic compounds [71,72]. Nevertheless, insect studies on polyphenol compounds are mainly related to the total content, and interference caused by proteins, amino acids, and peptides could cause an overestimation of these compounds [73,74]. Among other factors, both the total polyphenol content and quality profiles are highly variable between insect species (Table 2 and Table 3).

Even though little investigation has been conducted up to now, the presence of phenolic compounds in insects has been associated with their diet and with the ability of insects to synthesize phenolic compounds through the sclerotization process [16]. The influence of various feeds on the production of bioactive substances, such as polyphenols and flavonoids in *Protaetia brevitarsis* larvae, has been investigated [92]. The primary larvae feed (oak-fermented sawdust) was supplemented with fruits and vegetal (aloe, apple, banana, sweet persimmon, and sweet pumpkin). The authors found that compared with the mean total polyphenol content of the control group (19.2 mgGAE/g), the total polyphenol content of larvae receiving supplementary feeds was always higher; the combined mean from all groups receiving supplementary feed was 25.5 mg/g (the highest polyphenol content was observed in larvae receiving sweet pumpkin, 30.4 mg/g), whereas the total flavonoid contents in groups receiving supplemental feed do not vary significantly concerning control group. This suggests that diet can affect the uptake of polyphenols in insects as early as the larval stage [16]. In this study, the insects appear to have a selective uptake of flavonoids, mainly kaempferol and quercetin, as well as flavones, such as tricine and isovitexin. Most of these compounds have been identified in their glycosylated form with glucose, rhamnose, or galactose. Flavonols and flavones synthesized by the host plant are metabolized or absorbed by the insect. An understanding of an insect’s phenolic profile can provide indications of their possible use as functional foods.

## 2. Identification and Characterization of Phenolic Compounds in Insects

The European community categorizes insect-based food as “novel food” according to Reg. 2015/2283 [93]. A starting list of 12 species of insects is under evaluation; however, the first focus interest of the European Food Safety Authority (EFSA) is the risk control of edible insects. To date, only three insects (the house cricket *A. domesticus*, yellow mealworm *T. molitor*, and migratory locust *Locusta migratoria*) are officially retained edible insects; their trade has been approved by the European Commission after the positive scientific opinion of the EFSA [94,95,96]. Since regulation (EU) No. 2017/2470 allows the trade of insect-based products (either whole or in the form of a powder) (EU 217/2470) [97], the FAO recommended the development of analytical methods to verify the authenticity, quality, and safety of products of insect powders [10,98]. No specific indications are officially reported regarding the detection and assay of polyphenols; however, research of phenolic compounds derived from insect diets gained interest, especially combined with the exploration of insects’ feeding habits [17].

Literature data report the use of larvae or adult insects instead; pupae are rarely used [99,100]). When necessary (i.e., for crickets, locusts, and grasshoppers), wings, legs, and antennae are removed, while in scorpions, the stingers are eliminated [15]. Several procedures provide freeze-drying of insects followed by grinding them into powder before processing [22,99]. Sometimes, powdered insects are defatted with hexane washing, and the lipid-free solids are used for the extraction of water-soluble extracts [101]. In other cases, the defatting step is performed by microwave-assisted extraction (MAE) with petroleum ether, and the residues are collected for the next extraction step in alcoholic (methanol, ethanol) or alcoholic/aqueous solutions at a fixed temperature and time [14,16]. Ssepuuya et al. [15] report a study on the suitable conditions for the extraction of antioxidant compounds from *Ruspolia differens* and prove that the defatting step affects the amount of the antioxidant compounds. The antioxidants are predominately found in the apolar nonfat phase, and the highest concentration of antioxidant compounds is obtained after sonication for 60 min. Moreover, the authors demonstrated that the amounts of total phenols and flavonoids are relatively higher and comparable to those of many fruits and vegetables. Different, but at the same time very interesting, is the method proposed by Vasconcelos dos Santos et al. [102] that makes use of the crude oil (SRO) extracted from *Speciomerus ruficornis* larvae. The artisanal extraction provides that entire and wet larvae are directly placed on heat (150 °C) in a sealed stainless-steel pan until the oil flows out from the bugs, and the total polyphenol content is analyzed from SRO.

The quantification of bioactive compounds has obtained a great interest in food products, especially as relevant to preservation or to the prevention of deterioration by oxidation. Some antioxidants, such as polyphenols (e.g., caffeic acid, tannic acid, ferulic acid, ascorbic acid, and quercetin), became key compounds for neutralizing the damaging effects of oxidation [103]. A common methodology used for the quantification of antioxidant capacities is the Folin–Ciocalteu (FC) colorimetric method, which is based on the single electron-transfer (SET) reaction [41,78,104]. This assay was also adopted to evaluate the total polyphenol amount in edible insects or insect meal powder. However, the action mechanism of FC assay is centered on oxidation/reduction reaction, and it is prejudiced by the presence of other nonphenolic compounds, such as ascorbic acid, other enediols, and oleic acid [104]. This means that the total polyphenol content evaluated through FC assay reflects the oxidizing capacity of several different compounds together. Some researchers [101] reported the total polyphenol index (TPI) of water-soluble extracts of twelve commercially available edible insects and two invertebrates. Results are expressed as milligrams of gallic acid equivalents (GAE) per 100 g of defatted sample.

Crickets, grasshoppers, silkworms, African caterpillars, and evening cicadas displayed values of antioxidant capacity two- or three-fold higher in vitro than orange juice or olive oil [101]. However, the phenolic content of analyzed samples, as determined by TPI following a procedure modified by Giacintucci et al. [105], showed higher values only for grasshoppers compared to fresh orange juice. The authors suggested that the observed antioxidant capacity was not only due to the polyphenol compounds but also to the proteins, which is in agreement with the literature data [18,106].

Analytical instrumental techniques are currently adopted to identify and characterize peptides, proteins, or lipids differently extracted from insects [98], insect organs [84]), or hemolymph [107]. Indeed, it is well-known that standard analytical techniques are generally used for the identification and quantitative determination of phenolic compounds in several food matrices and also in insect meal powder. The coupling of liquid chromatography (LC) to mass spectrometry (LC-MS) facilitates metabolite identification and quantitation by reducing sample complexity and allowing analyte separation before detection [108]. Mass spectrometry has proven to be a very capable technique in flavonoid analysis because of its high sensitivity and signal reproducibility; moreover, the ability to couple with chromatographic techniques leads to both qualitative and quantitative determinations [109,110,111,112,113,114,115,116]. LC-MS and MS/MS are becoming the methods of choice for detecting analytes in complex samples. Seventeen phenolic compounds in edible *A. domesticus* were characterized and determined by HPLC-MS [16]. The authors have investigated the content of phenolic compounds in a commercial and organic sample, and major compounds identified in both extracts correspond to 4-hydroxybenzoic acid, *p*-coumaric acid, ferulic acid, and syringic acid. Several bioactive compounds, including phenolic acids and flavonoids, have been detected in the extract from the edible insect *P. vicina* [22]. The characterization of these components was carried out by HPLC-MS/MS. The most abundant identified antioxidant compounds are salicylic acid, gallic acid, liquiritigenin, and naringenin. The same authors also report the antioxidant activity of the analyzed extract and the characterization and quantification of fatty acids. Again, by mass spectrometry but with the help of other spectroscopic techniques, such as NMR, used to gain structural information for different classes of compounds [117,118,119,120], hydroxytyrosol derivatives were identified in the edible *Blaps rynchopetera* [26]. The insect *Blaps japanensis* is employed as an ethnomedicine in China for the treatment of several disorders, such as cancer and inflammation [121]. The authors analyzed the extract obtained from these insects and identified eight phenolic compounds, two of which were already known. The characterization was carried out by spectroscopic studies, such as NMR and high-resolution mass spectrometry. Liu et al. [14] investigated the antioxidant activity of an ethanolic extract of *H. parallela Motschulsky* a black beetle, a common crop pest in China. The authors characterized the extract by liquid chromatography and used it for identification of a standard solution of phenolic compounds: gallic acid, quercetin, catechin, protocatechuic acid, epicatechin, protocatechualdehyde, resveratrol, ferulic acid, and 4-hydroxyacetophenone [14]. Using a metabolomics approach, Fu et al. have identified more than 200 flavonoid metabolites in *Antheraea pernyi,* an edible insect; the study was conducted by LC-MS/MS in positive and negative modes. These flavonoid metabolites came from eight subclasses, including flavones, flavonols, flavonoids, flavanones, polyphenols, isoflavones, anthocyanins, and proanthocyanidins. The most abundant identified compounds are: hyperoside, isoquercitroside, tricine 7-*O*-hexoside, hesperetin 5-*O*-glucoside, and protocatechic acid [84].

## 3. Insects’ Bioactive Compounds as a Function of Diet

Polyphenols are vastly acknowledged as healthy substances, able to exert diverse bioactivities linked to chronic diseases, such as antioxidant, anti-inflammatory, and anticancer activity. Insects, however, are not capable to produce polyphenols de-novo and achieve them from their diet. Since the early 20th century, several studies have tried to relate insects’ herbivore feeding to their polyphenol content [17,77,88,122,123,124,125]. A recent study by Yoon et al. [92] also evidenced a similar aspect. Indeed, the authors ascertained that the content of nutrients and polyphenols of *P. brevitarsis* larvae could be tailored by supplementing auxiliary feeds, including aloe, apple, banana, sweet persimmon (*S. persimmon*), and sweet pumpkin (*S. pumpkin*). The feed and the breeding environment modification can be used to alter *P. brevitarsis* larvae’s capacity to serve as a healthy functional food. Haber et al. [99] report a study on the nutritional characterization of bees as a function of diet. They introduced two different diets: (i) bees fed only in nature, collecting pollen and nectar (natural diet) and (ii) bees fed in nature with the addition of a sucrose solution, as usually is performed by beekeepers during the winter. Total polyphenolic content is determined according to Musundire et al. [75] using the Folin–Ciocalteu assay. Reported data demonstrate that the nutritional value is found to differ between larvae and pupae, whereas antioxidant properties changed only between the different diets. A. domesticus was also subjected to study to verify the effect of diet on polyphenol content. In particular, the objective of Nino and coworkers’ study [16] was to elucidate the phenolic composition of farmed A. domesticus consuming two different diets (organic and commercial) and evaluate their potential in vitro antioxidant activity. The content of total phenolic compounds, determined by FC assay, revealed a high phenols content that cannot be attributed only to the phenolic compounds present in the sample but also to other components that are able to react with the Folin–Ciocalteu reagent. For example, the cricket extracts contained unsaturated fatty acids, vitamins, and free amino acids that have shown reactivity with the FC reagent in previous studies [74]. The confirmation of the presence of phenolic compounds in insects has been previously reported for a variety of species, mainly Lepidopterans (e.g., butterflies and moths) and also in this case, the main assumption is that these compounds are directly correlated with the insects’ diet [126].

## 4. Biological Activity of Polyphenols Extracts Obtained from Insects

### 4.1. Antioxidant Bioactivity

Currently, insect phenolics have mostly been assayed for their antioxidant bioactivity. However, kaempferol and quercetin found in insects can lead to other biological activities. Phenolic compounds decompose peroxide species, neutralize free radicals [127], and they can be able to bind to metal ions when the number and the specific location of hydroxyl groups in the molecule allow for it [128]. To investigate the antioxidant activity of phenolic compounds in insects, Liu et al. [14] evaluated the water extracts (WE) and ethanol extracts (EE) obtained from dark black chafer beetle (*H. parallela*). The EE had a superior activity compared to the butyl hydroxytoluene (BHT) standard, showing a better peroxidation inhibition activity. The WE revealed a higher reducing power than EE. Both extracts also proved to be a better chelating agent concerning ethylenediaminetetraacetic acid (EDTA) in sequestrating the iron ions. In EE, the authors also found a substantial catechin concentration (7.66 ± 0.05 mg/g extract).

Ethanolic extracts obtained from house cricket (*A. domesticus*) and mealworm (*T. molitor*) showed an in vitro antioxidant activity evaluated with the 1,1-diphenyl 2-picrylhydrazyl (DPPH) assay with around 80% inhibition [24].

Ssepuuya and coworkers [15] in recent research also evaluated the effects of the defatting procedure on *R. differens* (grasshopper) pastes. It has been found that the antioxidant activity of the nondefatted samples was similar to that of the defatted ones, suggesting that antioxidant compounds are located within the polar components of the pastes. This implies that the defatting step is not necessary to obtain a more active dietary supplement.

Pyo et al. [78] evaluated the bioactivities of six Korean edible insects (*Allomyrina dichotoma*-AD, *Tenebrio molitor*-TM, *Protaetia brevitarsis*-PB, *Gryllus bimaculatus*-GB, *Teleogryllus emma*-TE, and *Apis mellifera*-AM) to develop functional food ingredients. Ethanol extracts of AD were considered for antioxidation capacity, PB and AM for nitrite scavenging, and TE for anticoagulation, antioxidation, and hemolysis. Experimental data collected employing in vitro experimental tests showed the polyphenolic and flavonoidic content is not related to the measured antioxidant activity. Interestingly, even if not reported in the text, the major content of polyphenols has been found in GB and TE extracts. These were the only species fed by vegetable supplements.

Silkworm pupae (SWP) are another type of industry byproduct that can be recovered to exploit its nutritional properties to enhance the human diet. Sadat et al. [23], in their minireview, resume all the bioactive compounds so far identified in SWP methanolic extracts. It has been evidenced the presence of several polyphenols, including quercetin, resveratrol, kaempferol, myricetin, and naringenin. All these substances are accredited with strong antioxidant activities. This is also evident for the extracts of SWP (*Bombyx mori*, *Antheraea assamensis,* and *Antheraea mylitta*) that possess high ROS scavenging activity shown through by DPPH [129] (2,20-azino-bis-3-ethyl-benzothiazoline)-6-sulfonic acid (ABTS) and ferric reducing antioxidant power (FRAP) essays [130].

### 4.2. Other Biological Effects

Several biological effects linked to the presence of active substances have been evaluated in the literature (Table 4).

Zhang et al. [22] prepared a hydroethanolic extract of *P. vicina* to characterize its bioactivity. In particular, the pancreatic lipase (PL) inhibitory activity antioxidant activity and total flavonoid and total polyphenol contents of *P. vicina* extract have been tested in vitro. Phenolic acids such as salicylic acid and gallic acid and flavonoids such as liquiritigenin and naringenin were found. These constituted the major polyphenols in the *P. vicina* extracts. Docking studies evidenced an interaction of these four major constituents of the polyphenolic fraction with PL. Based on the measured antioxidant and PL inhibitory activities of this extract, a nutraceutical application to treat obesity and reduce oxidative stress-induced diseases can be advised.

Deori and coworkers [130] also evidenced that methanolic extracts of *A. assamensis* showed stronger antityrosinase activity of SWP as compared to kojic acid, while B. mori SWP extracts were characterized by antigenotoxic activity.

*B. japanensis* was the subject of a characterization study by Yan et al., [121]. This research group individuated new compounds called Blapsins (C to J) involved in several interesting bioactivities. Each compound was tested against cancer cells (A549, Huh-7, K562) and the COX-2 isoenzyme, ROCK1, and JAK3 genes. It was shown that the Blapsins (C to J) all have anticancer activity; in addition, Blapsin C, D, and F possess ROCK1 inhibitory activity, and Blapsins (C to G) have selective inhibitory activities versus the JAK3 family genes. All these activities show the possible beneficial effects of *B. japanensis* extract for the treatment of oncological, cardiovascular, and neurological disorders, organ transplantation, and autoimmune diseases. The study evidence that these results somewhat justify Chinese ethnomedicine.

Most of the reported studies evaluated the possible positive effects of the insect extracts on the human diet, often ignoring their adverse effects. However, it must be considered that these extracts can also contain anti-nutrients that should be removed or reduced. Kunatsa and coworkers [41] conducted a qualitative–quantitative screening of two edible insects: *Macrotermes facilger* and *Henicus whellani*. The extracts were characterized by antioxidant and antimicrobial properties, attributed to the polyphenolic content. However, oxalates, tannins, and cyanogenic glycosides were also found. These might have antinutritional properties; therefore, the authors suggest, food processing, such as boiling and cooking, to reduce antinutrient concentrations to safe levels before insect consumption.

### 4.3. Phenols’ Internal Synthesis and Immune Defences

On the other hand, some nondietary phenolic compounds that are found in insects’ bodies are derived from a chemical mechanism of sclerotization that leads to the phenols synthesized through the phenoloxidase enzyme [17,132].

In insects, melanins (eumelanin and pheomelanin) are synthesized for several purposes. These include color patterning, cuticle sclerotization, organogenesis, clot formation, and innate immunity. Traditional views of insect immunity detail the storage of prophenoloxidases inside specialized blood cells (hemocytes) and their release upon recognition of foreign bodies [133].

The pathway called the prophenoloxidase activating (proPO) system represents a defense and/or recognition system at first proposed for arthropods [134,135]. The proenzyme is converted to its active form, phenoloxidase, by proteolytic cleavage, and the resulting enzyme catalyzes both the o-hydroxylation of monophenols and the oxidation of diphenols to quinones. In turn, these quinones are polymerized nonenzymatically to melanin. This pigment is ubiquitous throughout the animal kingdom, and melanization supports hemocyte reactivity to foreign agents. Activation depends upon a cascade of serine proteases and other factors in the hemolymph, and some of these factors are sensitive to ß-l,3-glucans, lipopolysaccharides (LPS), or other carbohydrates derived from bacteria or from microbial cell walls; therefore, there are certain biochemical and functional similarities to the alternative pathway of complement. Phagocytosis, encapsulation, clotting, microbial killing, and wound repair are defense responses in which the component proteins of the proPO system are involved [135].

Reactive forms of oxygen, such as superoxide anion, hydroxyl radical, and hydrogen peroxide anion, have been implicated as components of vertebrate and invertebrate [136,137,138] cytotoxic mechanisms. The propensity of quinones for redox cycling makes these eumelanin precursors potential sources of the reactive forms of oxygen [139,140,141]. It has been demonstrated [142] that phenolic compounds exhibit cytotoxic activity toward human melanoma cells since they can be converted into toxic products by tyrosinase. Thus, the proPO system produces several molecules, including polyphenols and melanin capable of having a key role in specific bioactivities in insects, but their real beneficial impact on health has yet to be evaluated [143,144].

## 5. Final Remarks

This review shows the potential of insects as providers of a wide variety of bioactive compounds, such as polyphenols, which are widely recognized as health substances. Polyphenols are represented in insects because may function as pigment and chemical defense. Insects can selectively absorb and accumulate the flavonoids in their body from the larval stage via their host plant; therefore, the diet is the determinant for the type and amount of polyphenols present. They also may synthesize and store nondietary phenolic compounds through the sclerotization process. Currently, insect phenolics have mostly been assayed for their antioxidant bioactivity; however, they also exert other bioactivities, such as antiinflammatory and anticancer activity, antityrosinase, antigenotoxic, and pancreatic lipase inhibitory activities. Although several studies have been conducted on the use of insects, both whole and in flour form, as food supplements and as extracts in the treatment of acute diseases, there are few reports on the identification and characterization of the polyphenol profile. This is due to the most common adoption of unselective colorimetric methods as FC, and this may lead to an overestimation of phenolic content. Although an increasing number of studies focus on polyphenol profiling using mass spectrometry (LC-MS and MS/MS) techniques, to date, only fragmented data are available on only a few edible insect species.

Based on recent scientific developments on this topic, future research aims to encourage an investigation of new different classes of insects and polyphenols to evaluate their real beneficial health impact.

Despite these gaps, the high nutritional value as well as the presence of bioactive compounds associated with their undoubtedly ecological properties suggest insects as having a role in sustainable and functional foods. To date, only a few studies have evaluated biological, chemical, and allergenic risks and the presence of antinutrients. It should also be reminded that insects are typically processed by roasting, freezing, extrusion, and blanching, among other methods. Even if these processes do not significantly affect the total phenolic content in processed fruits and plants [17], up to date, there are not enough studies to describe the effects of such processing methods on insects. While actual knowledge could suggest that mild processing can maintain the functionality of these compounds, it is strongly advisable that processing time and temperatures should be evaluated and optimized. It is paramount to define standards, criteria, or guidelines for the management of insect-derived products. Indeed, legal regulations are the key prerequisite for the correct development of insect farming and the effective marketing of insect-based foods.

## Figures and Tables

**Table 1:** Environmental impact of mealworms and cricket compared to other animal products.

	Chicken	Pork	Beef	Insects	Reference
Water L/kg _meat_	2300	3500–22,000	43,000	40	[8,29]
Feed kg/kg live _animal_	2.5	5	10	1.7	[30]
Land ha/kg _protein_	2–3.5	2–3.5	10	1	[31]
CH_4_ emissions g/kg _biomass_	n.r ^1^	1.9	114	0.1	[32,33]
CO_2_ emissions g/kg _biomass_	n.r ^1^	79.6	285	7.6	[32,33]

^1^ n.r.: not reported.

**Table 2:** Total Polyphenol Index (TPI) expressed as gGAE/100 g (GAE, Gallic acid equivalent).

Insects	gGAE/100 g	Reference
Stink bugs (*Encosternum delegorguei*)	3.6	[17,75]
Cricket (*Henicus whellani*)	0.08	[17]
Cricket (*Henicus whellani*)	0.77	[27]
Beetle (*Eulepida mashona*)	0.08	[76]
Ground cricket (*Henicus whellani*)	0.77	[77]
Stinkbugs (*Encosternum delegorguei*)	3.6	[17,75]
House cricket (*Acheta domesticus*)	0.3–5.0	[24]
Chafer beetles (*Holotrichia parallela*)	5 g	[14]
Beetle (*Eulepida mashona*)	0.08	[27]
Rhinoceros beetle (*Allomyrina dichotoma)*	0.13	[78]
Mealworm beetle *(Tenebrio molitor)*	0.26	[78]
Scarabaeidae *(Protaetia brevitarsis)*	1.18	[78]
Cricket *(Gryllus bimaculatus)*	1.56	[78]
Cricket *(Teleogryllus emma)*	1.55	[78]
Bee *(Apis mellifera)*	1.24	[78]

**Table 3:** Polyphenolic compounds in different species of insects.

Family/Species	Polyphenolic Compounds	Reference
Black ant		
*Polyrhachis vicina*	Salicylic acid, trans-cinnamic acid, vanillic acid, isoferulic acid, gallic acid, 3,4-dihydroxybenzoic acid, formononetin, liquiritigenin, quercetin, caffeic acid, naringenin, catechin, sakuranetin, and L-epicatechin.	[22]
Acrididae		
*Acheta domesticus*	Quinic acid, gallic acid, 4-hydroxybenzoic acid, chlorogenic acid, caffeic acid, syringic acid, *p*-coumaric acid, ferulic acid, sinapic acid, 2-hydroxybenzoic acid, daidzein, quercetin, naringenin, and apigenin	[16]
*Dissoteira carolina*	Quercetin (3,3′,4′,5,7-pentahydroxyflavone) and quercetin-β-3-*O*-glucoside	[79]
*Schistocerca americana*	Luteolin (3′,4′,5,7- tetrahydroxyflavone) and β-3-*O*-glucoside	[80]
Beetle		
*Holotrichia parallela*	Resveratrol, 4-hydroxyacetophenone, protocatechualdehyde, ferulic acid, gallic acid, protocatechuic acid, epicatechin, quercetin, and catechin	[14]
Caterpillar		
*Rondotia menciana*	Quercetin-glycosides and kaempferol-glycosides	[81]
Silkworm		
*Bombyx mori*	Quercetin, kaempferol, and quercetin-glycosides	[82,83]
Butterfly		
*Antheraea pernyi*	Hyperoside (quercetin 3-*O*-glucoside), isoquercitroside, tricin 7-*O*-hexoside, hesperetin 5-*O*-glucoside, protocatechuic acid, luteolin 7-*O*-glucoside (cynaroside), kaempferol 3-*O*-glucoside (astragalin), *C*-hexosyl-luteolin *O*-*p*-coumaroylhexoside, luteolin 6-*C*-glucoside, tricin 4′-*O*-(β-guaiacylglyceryl) ether *O*-hexoside, orientin, luteolin *C*-hexoside, kaempferol 3-*O*-galactoside (trifolin), and tricin 4′-*O*-(β-guaiacylglyceryl) ether 7-*O*-hexoside ^1^	[84]
*Melanargia galathea*	Tricin (4′,5,7-trihydroxy-3′,5′-dimethoxyflavone), apigenin (4,5,7-trihydroxyflavone), tricin 7-glucoside, orientin (8-glucosylluteolin), luteolin 7-diglucoside, orientin 7-glucoside, vitexin 7-glucoside, isoorientin (luteolin 6-*C*-glucoside), isovitexin (6-*C*-glucosylapigenin), and tricin 4′-conjugate	[85]
*Coenonympha pamphilus*	Tricin (4′,5,7-trihydroxy-3′,5′-dimethoxyflavone)	[86]
*Lysandra coridon Poda*	Kaempferol-glycosides	[87]
*Polyommatus icarus*	Quercetin, kaempferol, and quercetin-glycosides and kaempferol-glycosides	[88]
*Pieris brassicae*	Kaempferol glycosides and ferulic and sinapic acids	[79]
*Melanargia galathea*	Glycosides of tricin (tricin-glycosides), lutexin, and tricin (4′,5,7-trihydroxy-3′,5′-dimethoxyflavone)	[89]
Hymenoptera		
*Amauronematus amplus, Arge* sp. *, Nematus bre-vivalvis, Nematus pravus, Nematus viridis, Nematus alpestris, Trichiosoma scalesii*	Quercetin-glycosides, kaempferol-glycosides	[90]
*Neodiprion sertifer*	(+)-Catechin 7-*O*-β-glucoside, isorhamnetin 3,7,4′-tri-*O*-β-glucosid, kaempferol 3,7, 4′-tri-*O*-β-glucoside, kaempferol 3,7, 4′-tri-*O*-β-glucoside, and quercetin 3,7,4′-tri-*O*-β-glucoside	[91]

^1^ More abundant out of a total of 225 identified compounds.

**Table 4:** Biological activity of insect extracts.

Insect Species	Most Abundant Molecules	Activity	References
*P. vicina*	salicylic acid, gallic acid, liquiritigenin	PL inhibitory activity	Zhang et al. [22]
*A. assamensis*	not specified	antityrosinase activity	Deori et al. [131]
*B. mori*	not specified	antigenotoxic activity	Deori et al. [131]
*B. japanensis*	blapsins	anticancer activity, inhibitory activities versus the JAK3 family genes; oncological, cardiovascular, and neurological disorders, organ transplantation, and autoimmune diseases	Yan et al. [121]
*Macrotermes facilger*	polyphenols	antioxidant and antimicrobial properties;	Kunatsa et al. [41]
*Macrotermes facilger*	oxalates, tannins, and cyanogenic glycosides	antinutritional properties	Kunatsa et al. [41]
*Henicus whellani*	polyphenols	antioxidant and antimicrobial properties;	Kunatsa et al. [41]
*Henicus whellani*	oxalates, tannins, and cyanogenic glycosides	antinutritional properties	Kunatsa et al. [41]

## Data Availability

Not applicable.
